# Effectiveness and Safety of Prochlorperazine in Indian Patients with Acute Vertigo: Results from a Large, Prospective, Post-marketing Observational Study

**DOI:** 10.1007/s12070-023-03831-0

**Published:** 2023-06-14

**Authors:** Mohan Kameswaran, M B Bharathi, Carlton Periera, Sudipta Chandra, Hari Krishna Reddy, Manjeeta Gupta, Deepa Sholapuri, Kartik Peethamabaran

**Affiliations:** 1 Madras ENT Research Foundation, Chennai, Tamil Nadu India; 2 JSS Hospital, Mysore, Karnataka India; 3 Bosco ENT Nursing and Research Centre, Mumbai, Maharashtra India; 4 Purnam Medicare, Kolkata, West Bengal India; 5 Ramya Hospital, Secunderabad, Telangana India; 6 Abbott Healthcare Pvt. Ltd, Mumbai, Maharashtra India; 7 Madras ENT Research Foundation, No-1, 1st Cross Street Off, 2nd Main Road, Raja Annamalaipuram, 600028 Chennai, Tamil Nadu India

**Keywords:** Acute vertigo, SVVSLCRE, Prochlorperazine

## Abstract

**Aim:**

To assess the efficacy and safety of prochlorperazine in Indian patients with acute vertigo.

**Methods:**

In this prospective, multicenter, open-label, post-marketing observational study, patients with acute peripheral vertigo of different etiologies received 5 mg prochlorperazine thrice a day for 5 days. The primary endpoints were percentage of patients with improvement in (1) vertigo symptoms and (2) clinical response as per scale for vestibular vertigo severity level and clinical response evaluation (SVVSLCRE) from baseline to end of treatment (Day 6). The key secondary endpoints were (1) improvement in nystagmus grading, and (2) safety and tolerability Efficacy of prochlorperazine by route of administration of first prochlorperazine dose (oral or intramuscular) was also assessed.

**Results:**

Of 1716 enrolled patients (mean [standard deviation, SD]) age (42.0 [12.95] years; 53.6% men), 57.4% were diagnosed with Meniere’s disease, followed by vestibular neuritis (17.4%), labyrinthitis (16.7%), or ear surgery (8.5%). In the overall population, 91.1% of patients showed improvement in clinical response per SVVSLCRE grading at Day 6 (p < 0.0001 vs. non-responders). Nystagmus grading was improved in 99.7% (of patients. No adverse drug reactions events were reported. Tolerability of prochlorperazine was rated as good, very good, and excellent by 43.6%, 32.9% and 20.7% of patients, respectively. Among patients with postoperative vertigo, 80.1% showed improvement in clinical response. In the intramuscular and oral subsets, 85.5% and 92.1% of patients showed improved clinical response, respectively.

**Conclusion:**

Prochlorperazine showed improvement in severity of symptoms and clinical response in all subsets of vertigo patients, with a good safety and tolerability profile.

**Trial Registration Number:**

CTRI/2022/01/039287.

**Date of Registration:**

10 January 2022.

## Introduction

Vertigo refers to the perception of movement in the absence of actual physical movement, either in the self or in the environment [1]. As a symptom of peripheral or central vestibular dysfunction, vertigo is a common complaint in primary and emergency care [2]. Vertigo has a prevalence ranging from 10 to 30% population worldwide and an incidence of 1.4% each year; it affects about 15% to over 20% of adults yearly [3–5]. The prevalence of vertigo increases with age and is 2–3 times more common in women than in men [2].

A multicenter study from India reported that in 74% of patients with vertigo, the etiology was peripheral [6]. In the Indian context, another study reported that 5% of all patients visiting a general physician and about 10% of patients visiting neurologists and otorhinolaryngologists present with vertigo [7].

Vestibular suppressants constitute the mainstay of treatment for vertigo [8]. Prochlorperazine, is a vestibular suppressant, reduces vertigo by blocking the H1 receptor in the vestibule and the brain. It also has anti-dopaminergic (blocks the D2 receptors), anti-serotonergic (blocks 5HT3 receptors), anticholinergic and blocks alpha 1 receptors as well. It reduces the nausea and vomiting sensation by blocking chemoreceptor trigger zone (CTZ), vomiting center and D2 receptors [8, 9]. Additionally it due to its action on serotonergic neurotransmitter system (5HT3), it is a preferred choice for short-term symptomatic treatment of vertigo associated with anxiety. Prochlorperazine alleviates both vestibular and related vegetative vertigo symptoms such as nausea, vomiting and anxiety. Due to its comprehensive action, it is often the most frequently recommended drug for acute vertigo episodes [8–10].

While there is data available for effectiveness of dizziness with prochlorperazine, clinical studies assessing the effectiveness of prochlorperazine in reducing severity and frequency of acute vertigo of varied etiologies is yet to be established in India. Therefore, the objective of the current post-marketing observational study was to assess the effectiveness of prochlorperazine in the treatment of Indian patients with acute vertigo of varied etiologies.

## Materials and methods

### Study Design

This was a prospective, multi-center, open-label, single-arm, post-marketing observational study conducted at 25 Otorhinolaryngology departments across different cities of India. Based on the severity of vertigo, all eligible patients were prescribed prochlorperazine 5 mg () for a duration of 3 days or 5 days or prochlorperazine 12.5 mg intramuscular injection as per the investigator’s discretion and were followed up to 15 days.

The study was performed according to the principles of International Council for Harmonization of Technical Requirements for Pharmaceuticals for Human Use - Guideline for Good Clinical Practice, Declaration of Helsinki, and in compliance with the “The New Drugs and Clinical Trial Rules- 2019”, of the Ministry of Health, Government of India.

The trial was prospectively registered on Clinical Trials Registry – India on 10 January 2022 (CTRI registration number CTRI/2022/01/039287). The study protocol and related documents were reviewed and approved by the respective institutional review boards at each site before study initiation. Written informed consent was obtained from every patient before enrolment into the study.

### Eligibility Criteria

Patients of either sex aged 18–65 years who had been diagnosed with acute peripheral vertigo (Meniere’s disease, vestibular neuritis, labyrinthitis, or postoperative) were included in the study.

Patients diagnosed with BPPV, patients with history of psychiatric illness, cardiovascular, kidney or liver disorders, or hypersensitivity to phenothiazine derivatives, patients on antipsychotics or antidepressants, patients requiring hospitalization, patients with suspected or established subcortical brain damage, with or without hypothalamic damage, and pregnant or nursing women or those of childbearing potential not practicing reliable contraceptive methods were excluded.

### Study Endpoints

The primary endpoints of the study were (1) frequency and percentage of patients achieving improvement in vertigo symptoms using the scale for vestibular vertigo severity level and clinical response evaluation (SVVSLCRE) [11] from baseline to Day 6 and (2) frequency and percentage of patients achieving improvement in clinical response using SVVSLCRE at Day 6 .

The secondary endpoints were (1) frequency and percentage of patients experiencing change in severity of vertigo symptoms by nystagmus grading [12] from baseline to Day 6; (2) frequency and percentage of patients with reported adverse drug reactions (ADRs) and extrapyramidal symptoms from baseline to end of study visit on Day 15; (3) frequency and percentage of patients showing tolerability to prochlorperazine on Day 6; and (4) frequency and percentage of patients achieving relief from vertigo-associated symptoms from baseline to Day 6.

Exploratory endpoints included frequency and percentage of (1) postoperative vertigo patients achieving improvement in vertigo symptoms using SVVSLCRE and nystagmus grading from baseline to Day 6; (2) postoperative vertigo patients achieving improvement in clinical response using SVVSLCRE at Day 6; (3) acute vertigo patients achieving improvement in vertigo symptoms and clinical response using the SVVSLCRE by route of administration of first dose from baseline to Day 6; (4) acute vertigo patients achieving improvement in vertigo symptoms using nystagmus grading by route of administration of first dose from baseline to Day 6; (5) postoperative vertigo patients with ADRs and extrapyramidal symptoms (EPS) from baseline to end of study visit on Day 15; (6) postoperative vertigo patients showing tolerability to prochlorperazine at Day 6; (7) acute vertigo patients with ADRs and extrapyramidal symptoms by route of administration of first dose from baseline to end of study on Day 15; and (8) acute vertigo patients showing tolerability to prochlorperazine by route of administration of first dose based on self-reported questionnaire at Day 6.

### Study Assessments

Demographic details such as age, sex, height, weight, body mass index (BMI) and socioeconomic status were collected at baseline. Vestibular system examination at baseline included head thrust test, subjective visual vertical test, Fukuda stepping test, Romberg test, and past pointing and falling test.

The SVVSLCRE [11] was used to assess vertigo symptoms and their severity. Symptom severity was graded as Level I (scores 0–2) = absent vertigo/very mild, Level II (scores 3–4) = mild, Level III (scores 5–6) = moderate, Level IV (scores 7–8) = severe, or Level V (scores 9–10) = very severe vertigo. Clinical response was evaluated based on change in severity of vertigo symptoms from baseline to Day 6 and reported as worsening (increase by one level), no change, moderate (if levels changed from V to IV, IV to III, III to II, or II to I), good (if levels changed from V to III, IV to II, or III to I), very good (if levels changed from V to II or IV to I), or excellent (if level changed from V to I).

Using Alexander’s law, nystagmus was graded as Grade 1 if nystagmus was induced only by looking to the direction of the fast component i.e., opposite to the affected ear, Grade 2 if looking straight forward caused nystagmus, or Grade 3 if severe nystagmus was induced even by looking in the direction of the affected ear.

The severity of vertigo-associated symptoms (faintness, headache, hearing loss, nausea, vomiting, and tinnitus) was evaluated on a 4-point scale, where 4 = excellent, 3 = good, 2 = fair, and 1 = poor. Vertigo associated symptoms were assessed at baseline and Days 2, 4, 6, and 15. EPS such as acute dystonia, akathisia, tardive dyskinesia, Parkinsonism, akinesia, and neuroleptic malignant syndrome were recorded along with any ADRs from baseline to EOS visit on Day 15.

Tolerability was assessed based on reporting of overall health by patient as –poor, satisfactory, good, very good, or excellent.

### Statistical Analysis

Assuming a confidence level of 0.95, expected proportion of 0.8, and precision of 0.02 for the primary endpoint on patients achieving relief in acute vertigo based on the SVVSLCRE from baseline to EOS (Day 15) and a dropout rate of 10%, a sample size of 1708 patients was determined.

Enrolled set (ES) consisted of all patients recorded in the clinical database. Intention-to-treat (ITT) set consisted of all patients in the ES who had at least one follow-up visit post initiation of prochlorperazine treatment. Per protocol (PP) set consisted of all patients in the ITT set who were followed up to 15 days post-initiation of prochlorperazine treatment and who did not have any major protocol deviations.

Statistical analyses in this study were descriptive. Continuous data were summarized as mean and standard deviation (SD), and categorical variables were summarized as frequency and percentages. Demographics and other baseline characteristics, effectiveness analyses, and safety/tolerability analyses were performed using the ES as there was no difference between the ITT and PP sets.

## Results

### Demographics and Baseline Characteristics

**Fig. 1:**
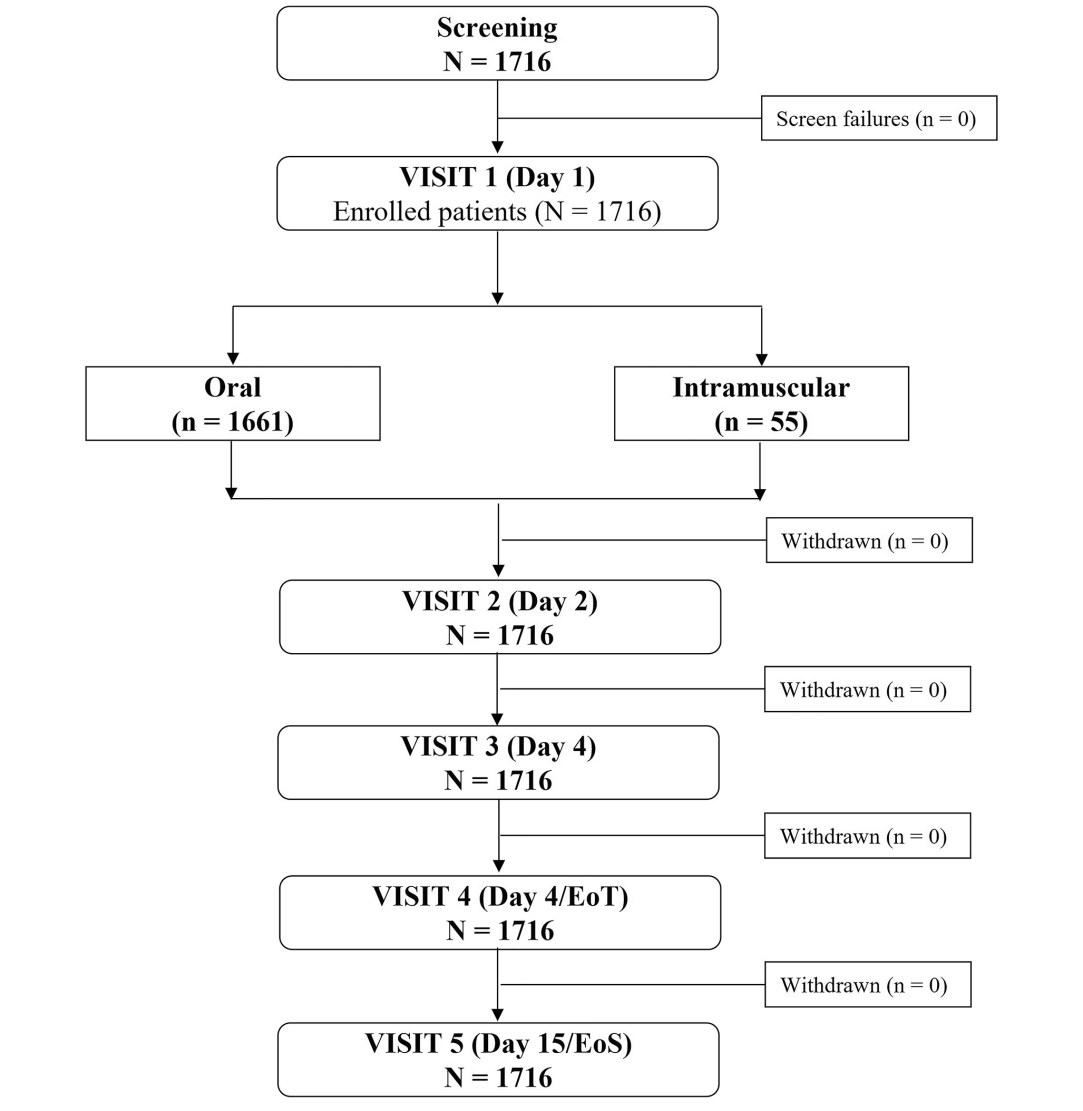
Study patients disposition chart

In all, 1716 patients (919 [53.6%] men and 797 [46.4%] women) with acute vertigo were enrolled in the study, all of whom completed the end of treatment visit on Day 6 (Fig. 1).

The mean (SD) age was 42.0 (12.95) years. Etiology of vertigo was Meniere’s disease in 57.4% patients, vestibular neuritis in 17.4%, labyrinthitis in 16.7%, and postoperative vertigo in 8.5% of patients (Table 1).

**Table 1:** Demographics and baseline characteristics

Characteristics	Prochlorperazine (N = 1716)
Age (years), mean (SD)	42.0 (12.95)
BMI (kg/m^2^), mean (SD)	24.6 (3.73)
Male sex, n (%)	919 (53.6)
Occupation, n (%)	
Professionals	221 (12.9)
Skilled workers/shop and market sales workers	201 (11.7)
Elementary occupation	166 (9.7)
Others*	734 (42.7)
Unemployed	394 (23.0)
Diagnosis, n (%)	
Meniere’s disease	985 (57.4)
Vestibular neuritis	298 (17.4)
Labyrinthitis	287 (16.7)
Postoperative vertigo	146 (8.5)
Surgical procedures for post-operative patients, n (%)	**N = 146**
Tympanoplasty	86 (58.9)
Stapedectomy	46 (31.5)
Otoplasty	13 (8.9)
Myringoplasty	1 (0.7)
Positive vestibular system examination results, n (%)	
Head thrust test	454 (26.5)
Fukuda stepping test	338 (19.7)
Subjective visual vertical test	289 (16.8)
Romberg test	211 (12.3)
Past pointing and falling test	110 (6.4)

*Clerks, craft and related trade workers, legislators/senior officials/manager, plant and machine operators/assemblers, skilled agricultural/fishery workers, skilled workers/shop and market sales workers, technicians/associate professionals

BMI, body mass index; SD, standard deviation

Out of 146 patients who experienced postoperative vertigo, tympanoplasty, stapedectomy, otoplasty, and myringoplasty were performed on 37.7% (55/146), 31.5% (46/146), (13/146) 8.9% and (1/146) 0.7%, respectively. Common comorbidities in the study population were hypertension (2.6%) followed by diabetes (1.2%) and hypercholesterolemia (0.6%). According to investigator discretion, 1632 of the 1716 patients were prescribed prochlorperazine for 5 days, while 84 were prescribed prochlorperazine for 3 days. Out of 146 post-operative patients, route of administration of the first dose was oral for 135 patients and intramuscular for 11.

### Effectiveness of Prochlorperazine in Patients with Acute vertigo

#### Outcomes in Overall Population

**Fig. 2:**
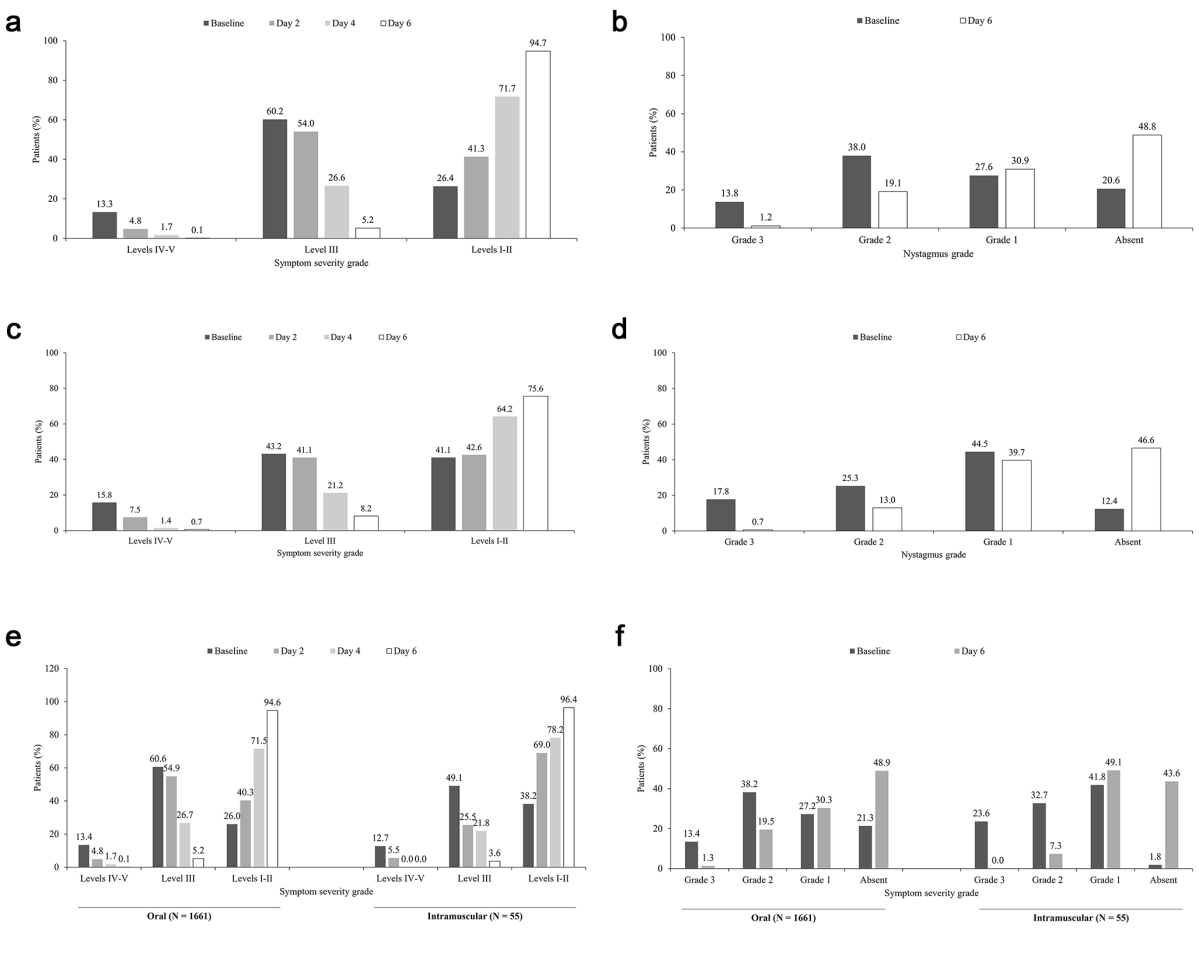
Effectiveness of prochlorperazine in improvement of vertigo symptoms. **A**) Symptom severity grading by SVVSLCRE in overall population. **B**) Nystagmus grading in overall population. **C**) Symptom severity grading by SVVSLCRE in patients with postoperative vertigo. **D**) Nystagmus grading in patients with postoperative vertigo. **E**) Symptom severity grading by SVVSLCRE in patients stratified by route of administration of first dose. **F**) Nystagmus grading in patients stratified by route of administration of first dose

In the overall study population, proportion of patients with moderate, severe or very severe symptoms (Levels III-V) as assessed using the SVVSLCRE gradually decreased and those with mild or no symptoms (Levels I-II) increased (Fig. 2A). At Day 6, 94.7% patients had severity levels I-II, 5.2% had severity level III, and only 0.1% had severity levels IV-V. Out of 1716 enrolled patients, 26.2%, 58.0% and 91.1% of patients achieved improvement in symptoms by Days 2, 4, and 6, respectively (Table 2). Moreover, 91.9% of the patients showed improvement in clinical response per SVVSLCRE grading by Day 6 (p < 0.0001 for difference between responders and non-responders; Table 3).

**Table 2:** Patients achieving improvement in vertigo symptoms per SVVSLCRE grading

Patient groups, n (%)	Improvement in SVVSLCRE grade^a^		
	Day 2	Day 4	Day 6
Overall population (N = 1716)	449 (26.2)	996 (58.0)	1564 (91.1)
Post-operative vertigo patients (n = 146)	34 (23.3)	79 (54.1)	117 (80.1)
Route of administration of first dose			
Oral (n = 1661)	426 (25.6)	963 (58.0)	1519 (91.5)
Intramuscular injection (n = 55)	23 (41.8)	33 (60.0)	45 (81.8)

^a^One level improvement in the SVVSLCRE scale was considered as improvement

SVVSLCRE, scale for vestibular vertigo severity level and clinical response evaluation

**Table 3:** Clinical response with prochlorperazine treatment using SVVSLCRE by Day 6

	Clinical response using SVVSLCRE by patient groups			
**n (%)**	All patients (N = 1716)	Postoperative vertigo (N = 146)	Route of administration of 1st dose	
			Oral (N = 1661)	Intramuscular injection (N = 55)
Worsening	1 (0.1)	-	1 (0.1)	0
No change	139 (8.1)	29 (19.9)	131 (7.9)	8 (14.5)
Moderate	863 (50.3)	52 (35.6)	837 (50.4)	26 (47.3)
Good	508 (29.6)	45 (30.8)	493 (29.7)	15 (27.3)
Very good	200 (11.7)	20 (13.7)	194 (11.7)	6 (10.9)
Excellent	5 (0.3)	-	5 (0.3)	0
***P value*** ^***a***^	< 0.0001	0.4666	0.4923	

^a^*P* value by chi square test for difference between responders (patients with moderate, good, very good, or excellent improvement) and non-responders (patients with worsening or no change)

Excellent = shift from Level V to I; Very good = shift from Levels V to II or IV to I; Good = shift from Levels V to III, IV to II, or III to I; Moderate = shift from Levels V to IV, IV to III, III to II, or II to I; No change = Levels V to V, IV to IV, III to III, II to II, or I to I; Worsening = all other shifts

SVVSLCRE, scale for vestibular vertigo severity level and clinical response evaluation

With respect nystagmus grading, proportion of patients with grade 3 nystagmus decreased from 13.8% at baseline to 1.2% by Day 6, and that with absent or grade 1 nystagmus increased from 48.3% at baseline to 79.7% by Day 6 (Fig. 2B). Moreover, 99.7% patients showed improvement in severity of symptoms by Day 6 as determined by nystagmus grading.

By the end of the treatment on Day 6, 97.8%, % 97.1%, 96.3%, 96.6%, 95.8%, and 97.8% of patients achieved relief in vertigo-associated symptoms faintness, headache, hearing loss, nausea, vomiting, and tinnitus, respectively (Table 4).

**Table 4:** Relief in vertigo-associated symptoms in overall population

Symptom, n (%)	Day 2	Day 4	Day 6
Faintness	1638 (95.5)	1636 (95.3)	1679 (97.8)
Headache	1617 (94.2)	1607 (93.6)	1667 (97.1)
Hearing loss	1571 (91.6)	1595 (92.9)	1652 (96.3)
Nausea	1606 (93.6)	1622 (94.5)	1657 (96.6)
Vomiting	1582 (92.2)	1593 (92.8)	1644 (95.8)
Tinnitus	1631 (95.0)	1649 (96.1)	1679 (97.8)

#### Outcomes in Patients with Postoperative vertigo

Among patients with postoperative vertigo (n = 146), 75.6% patients had severity levels I-II, 8.2% had severity level III, and only 0.7% had severity levels IV-V at Day 6 (Fig. 2C), and 23.3%, 54.1% and 80.1% of patients achieved improvement in symptoms by Days 2, 4, and 6, respectively. Clinical response per SVVSLCRE grading was observed in 80.1% by Day 6 (Table 2), but the difference between responders and non-responders was not significant (p = 0.4666; Table 3).

Proportion of patients with grade 3 nystagmus decreased from 17.8% at baseline to 0.7% by Day 6, and that with absent or grade 1 nystagmus increased from 47.2% at baseline to 71.6% by Day 6 **(**Fig. 2D**)**. All patients with postoperative vertigo showed improvement in severity of symptoms by Day 6 as determined by nystagmus grading.

#### Outcomes in Patients Stratified by Route of Administration of First dose

Route of administration of first dose of prochlorperazine was oral in majority of the patients (96.8%). Effectiveness outcomes as determined by SVVSLCRE and nystagmus grading were similar regardless of route of administration of first dose (Fig. 2E-F). Clinical response as determined by SVVSLCRE grading was achieved by 92.1% of patients in the oral subset and 85.5% of patients in the intramuscular subset by the end of the treatment on Day 6 (p = 0.4923; Table 3).

### Safety and Tolerability of Prochlorperazine in Patients with Acute vertigo

**Fig. 3:**
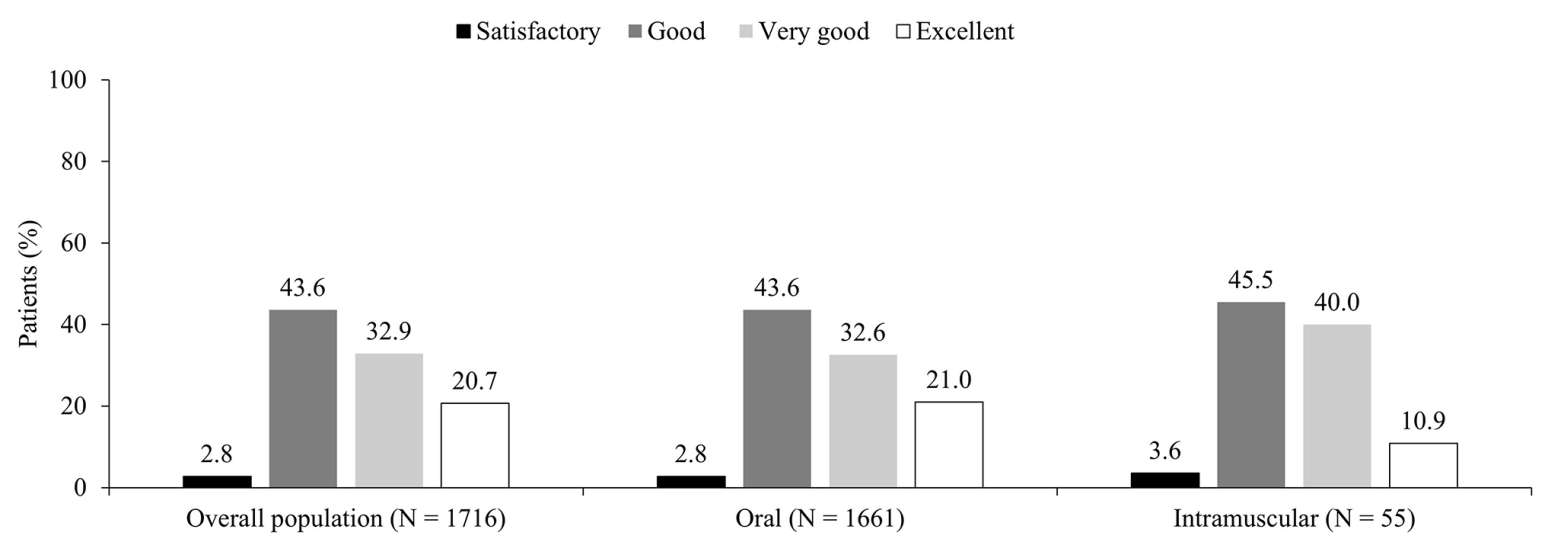
Tolerability of prochlorperazine

In the current study, no treatment-emergent ADRs or EPS were observed. By the end of treatment on Day 6, 43.6%, 32.9%, and 20.7% of patients reported good, very good, and excellent tolerability, respectively. Similar trends were observed in patients stratified by route of administration of first dose of prochlorperazine (Fig. 3).

## Discussion

The current study was designed to evaluate the effectiveness and safety of prochlorperazine in Indian patients with acute vertigo of varied etiologies. We found that by the end of treatment (Day 6), 91.9% (1576) out of 1716 patients showed significant improvement in clinical response as measured by SVVSLCRE grading.

In a study by Haldipur et al. where 500 patients with dizziness were administered prochlorperazine 5 mg three times a day, statistically significant change in the frequency of dizziness episodes from baseline at Week 1 was reported [13].

Our findings were in accordance with the study conducted by Kameswaran et al. where the authors compared clinicoetiological patterns and pharmacotherapy practices among Indian patients with vertigo. Prochlorperazine prevented recurrence of vertigo episodes in a significantly higher proportion of patients (76.6%) than betahistine (62.9%) and cinnarizine (57.6%) within first week (p < 0.0001) irrespective of gender or etiology of vertigo. [10].

Postoperative vertigo after ear surgery is common and can create considerable problems regarding management of patients and outcome of the surgical procedure. In a study by Weinmann et al., 45.8% participants experienced new vertigo after unilateral cochlear implant treatment, and a vestibular origin was suspected in 72.7% [14]. In the present study, 146 patients had postoperative vertigo, out of which 58.9% had undergone tympanoplasty, 31.5% had undergone stapedectomy, 8.9% had otoplasty and 0.7% had undergone myringoplasty. The improvement in vertigo symptoms in these patients was similar to that of total population, with 80.1% achieving clinical response by day 6 and all patients achieving improvement in nystagmus grade.

Among the mainstay of therapies available for management of acute vertigo, prochlorperazine is one of the few drugs available for both intramuscular and oral administration. The oral route has slower onset of action (20–30 min) in comparison with the intramuscular route (10–20 min) [15]. However, despite of this difference, this study did not report a huge difference between effectiveness of the two formulations in acute vertigo. In our study by the end of the treatment (Day-6) acute vertigo patients reported almost similar tolerability level between good, very well and up to excellent in both intramuscular and oral subset. No statistical significance difference was observed between two subsets with respect to tolerability to prochlorperazine based on self-reported questionnaire. Thus, intramuscular route of administration deployed in emergency care with careful supervision can be effective and well-tolerated.

In the current study, prochlorperazine has demonstrated notable safety profile with no extra pyramidal symptoms reported with both oral and injectable doses.

In the study by Haldipur et al. where 500 patients with dizziness were studied administered 5 mg of prochlorperazine thrice a day, During the course of the study, incidence of ADRs was 0.006% Observed ADRs were headache, asthenia, and somnolence, which were presumably attributed to prochlorperazine, and all of these were minor in severity and resolved [13].

Our study had a few limitations such as open label design, absence of control group; and lack of randomization. Despite these limitations, we had a large sample size, which was reflective of the real-world scenario across India. Further well-designed studies will be helpful in this regard.

## Conclusion

This study was conducted across 25 centers in India where prochlorperazine was found to be effective and well-tolerated in acute vertigo patients of varied etiologies. It was associated with significant improvement in the severity of symptoms and clinical response across different subsets of vertigo patients. Both oral and intramuscular formulations were effective and well-tolerated.
